# Longitudinal *in vivo* imaging of adult *Danionella cerebrum* using standard confocal microscopy

**DOI:** 10.1242/dmm.049753

**Published:** 2022-12-22

**Authors:** Pui-Ying Lam

**Affiliations:** ^1^ Neuroscience Research Center, Medical College of Wisconsin, 53226 Milwaukee, WI, USA; ^2^ Department of Cell Biology, Neurobiology and Anatomy, Medical College of Wisconsin, 53226 Milwaukee, WI, USA

**Keywords:** *Danionella cerebrum*, Adult, Imaging, Macrophages, Microglia, Endothelial cells

## Abstract

*Danionella cerebrum* is a new vertebrate model that offers an exciting opportunity to visualize dynamic biological processes in intact adult animals. Key advantages of this model include its small size, life-long optical transparency, genetic amenability and short generation time. Establishing a reliable method for longitudinal *in vivo* imaging of adult *D. cerebrum* while maintaining viability will allow in-depth image-based studies of various processes involved in development, disease onset and progression, wound healing, and aging in an intact live animal. Here, a method for both prolonged and longitudinal confocal live imaging of adult *D. cerebrum* using custom-designed and 3D-printed imaging chambers is described. Two transgenic *D. cerebrum* lines were created to test the imaging system, i.e. *Tg(mpeg1:dendra2)* and *Tg(kdrl:mCherry-caax).* The first line was used to visualize macrophages and microglia, and the second for spatial registration. By using this approach, differences in immune cell morphology and behavior during homeostasis as well as in response to a stab wound or two-photon-induced brain injury were observed in intact adult fish over the course of several days.

## Teaser

**Editor's choice:** Experimental imaging setup, materials and procedures for *in vivo* and longitudinal confocal imaging of the transparent adult vertebrate model *Danionella cerebrum.*

## INTRODUCTION

*Danionella cerebrum*, not to be confused with one of the other four *Danionella* species *Danionella translucida* (Britz et al., 2021), has recently been described as a genetically tractable vertebrate brain (Schulze et al., 2018) and neuroscience model (Penalva et al., 2018 preprint). *D. cerebrum* adults are, for the most part, optically transparent and have a body length of ∼12 mm. They are among the smallest known vertebrates, have the smallest adult vertebrate brain (Schulze et al., 2018) and only have minor dorsal ossification in the skull (Britz and Conway, 2016; Britz et al., 2021; Schulze et al., 2018). Whereas the larval zebrafish (*Danio rerio*) is an excellent model for *in vivo* imaging, juvenile and adult forms are less optically accessible. Internal organs and tissues, such as the central nervous system, are challenging to image within adult zebrafish due to pigmentation of the skin and ossification of the skeletal system, including the skull. Zebrafish pigmentation mutants, such as *casper* (White et al., 2008) and *crystal* (Antinucci and Hindges, 2016), represent a breakthrough for imaging of structures immediately below the skin in adults. Although essentially transparent, *casper* and *crystal* fish retain some opacity and their size at adulthood renders imaging of deeper structures challenging. As a viable alternative, *D. cerebrum*, with its small size and superior transparency, has been touted as a valuable animal model for adult-onset diseases, as well as for studies in developmental and evolutionary biology (Rajan et al., 2022). An annotated hybrid genome assembly of *D. cerebrum* is publicly available (Kadobianskyi et al., 2019). Genome modification techniques commonly used in zebrafish, such as Tol2-mediated transgenesis and CRISPR-Cas9-mediated gene editing, have been successfully applied to *D. cerebrum* (Schulze et al., 2018).

*D. cerebrum* is one of the most optical accessible adult vertebrate models. Live imaging of internal tissues, such as the brain (Ellett et al., 2011; Schulze et al., 2018) and blood vessels (Penalva et al., 2018 preprint), has been possible by using two-photon (2P)-microscopy in intact adult animals. Whereas larval *D. cerebrum* are very similar in size to larval zebrafish, sexually mature *D. cerebrum* are approximately three times shorter. A calculation of the volume scale factor shows that adult *D. cerebrum* have a volume that is ∼27 times smaller than that of adult zebrafish. This small size, combined with their natural transparency, facilitates the use of adult *D. cerebrum* for imaging deeper tissues and cells by using conventional confocal or multi-photon microscopy. To fully realize the potential of using *D. cerebrum* for long-term biological studies, a robust, repeatable and reliable method to perform longitudinal time-lapse imaging of adults is essential*.* As is the case with adult zebrafish, imaging of adult *D. cerebrum* while using standard anesthesia methods is limited to only a few minutes before survival is affected. Long-term imaging of adult zebrafish can be achieved when the animal is intubated (Castranova et al., 2022; Cox et al., 2018; Xu et al., 2015). Building upon this approach, specifically designed imaging chambers for adult *D. cerebrum* were designed, which allow for intubation, are flexible with respect to positioning the animal and are compatible for use with inverted as well as upright microscopes.

*In vivo* imaging of adult *D. cerebrum* has been difficult, primarily, because they do not tolerate standard methods used for imaging larval stages. Unlike larval fish, adults require ventilation by circulating water through the mouth and over the gills in order to maintain viability. Short-term live imaging of adult *D. cerebrum* has previously been demonstrated by anesthetizing fish with Tricaine-S at low doses and immobilizing them by partially embedding them in low-melt agarose (Schulze et al., 2018). Although adult *D. cerebrum* can tolerate being anesthetized for short periods of time, a lengthy exposure to Tricaine-S leads to reduced recovery rates. In light of this constraint and in an effort to avoid altering biological processes that are sensitive to anesthesia (Dong et al., 2021; Hristovska et al., 2020; Liu et al., 2019; Sun et al., 2019), a method that would allow for stable and repeatable positioning of adult fish for long-term longitudinal *in vivo* imaging was devised. This was achieved by completely encasing the fish in agarose while maintaining ventilation through an intubation tube. Mounting and positioning of adult *D. cerebrum* was facilitated with the use of a custom-designed and 3D-printed imaging chamber. These imaging chambers are suitable for inverted as well as upright microscopes. By using this method, adult fish can be imaged for several hours with no anesthesia, remain viable and physically healthy, and can be remounted and imaged repeatedly over several days, weeks or even months. In proof of principle experiments, the use of adult *D. cerebrum* as a model for brain injury and hemorrhage was demonstrated when using this mounting and imaging approach.

## RESULTS

**Fig. 1.**
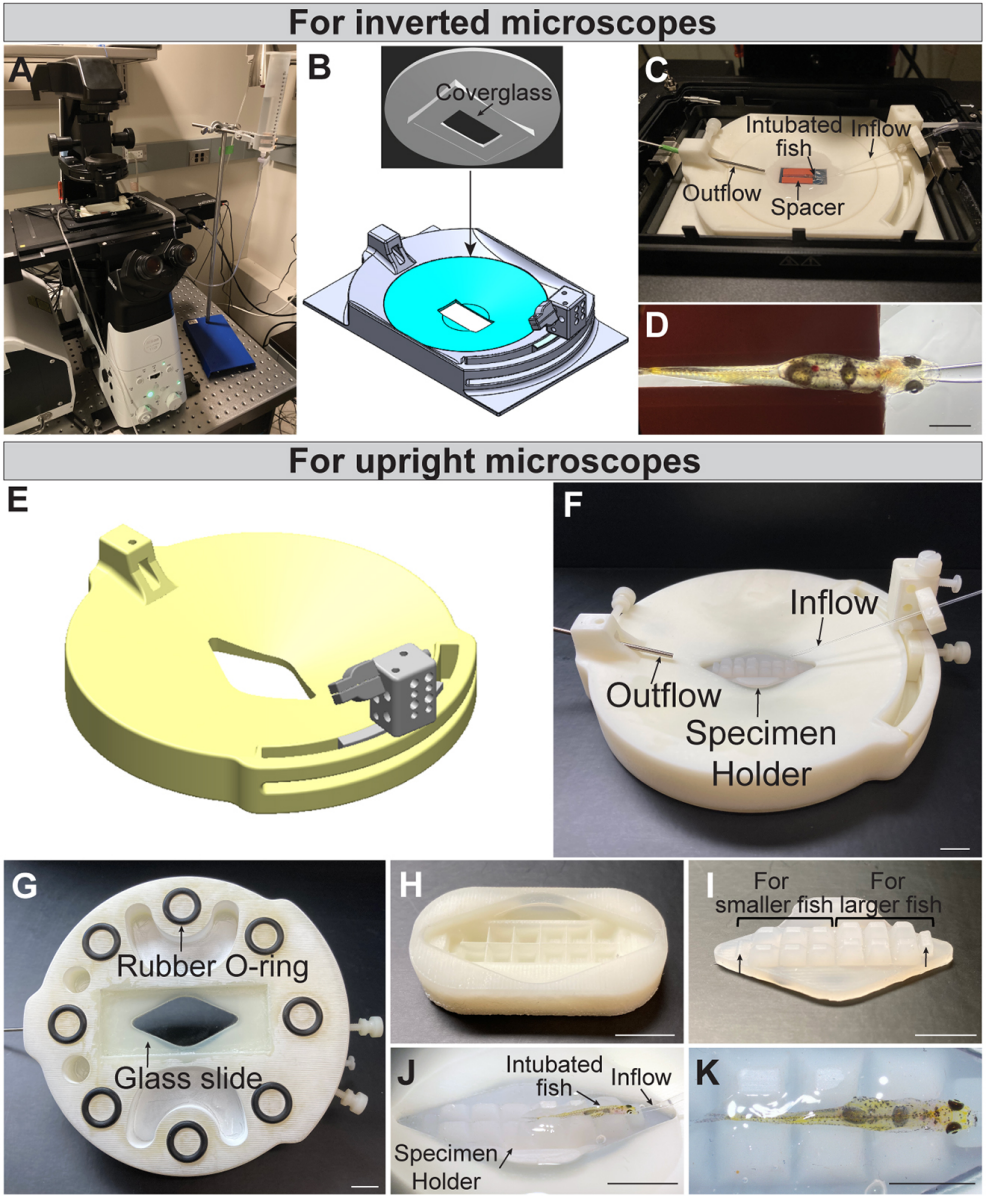
**Imaging chambers for long-term live imaging of awake adult *D. cerebrum.*** (A) Photograph of the *D. cerebrum* imaging chamber showing the system on an inverted microscope with elements designed to intubate adult fish. (B) Schematic showing the insert portion (blue) of the chamber with a coverslip attached to the bottom. This insert fits into the platform part, having a standard 96-well plate footprint. (C) Magnified view of the chamber showing the inflow tube, outflow tube and the spacer used for positioning the fish. Fish are mounted under 4% low-melt agarose. (D) An intubated and agarose-embedded fish positioned for imaging the brain. Scale bar: 2 mm. (E) Schematic of the chamber for an upright microscope. (F) Photograph of the imaging chamber, detailing the inflow tube, outflow tube and the specimen holder used to position the fish in upright orientation. Scale bar: 1 cm. (G) Underside of the imaging chamber. The glass slide allows for bottom illumination to assist fish placement on the microscope stage. The rubber O-rings prevent the chamber from slipping on the microscope stage. Scale bar: 1 cm. (H) Mold used to cast the silicone specimen holder shown in (F, I-K). Scale bar: 1 cm. (I) Magnified view of the silicone specimen holder shown in F. The spacer is wedge-shaped to assist in positioning the fish in an upright position before embedding it in agarose. One end of the specimen holder has shorter wedges to accommodate smaller fish. Arrows indicate head rest positions for the fish. Scale bar: 1 cm. (J) Angled side view of an intubated and agarose-embedded fish in a dorsal mount position using the shorter wedge side of the specimen holder as shown in I. Scale bar: 1 cm. (K) Dorsal view of the fish shown in J. Scale bar: 0.5 cm.

Methods for confocal live imaging of highly motile immune cells in zebrafish larvae have previously been described (Lam et al., 2014). Although the imaging methods for larval zebrafish can be applied directly when imaging larval *D. cerebrum*, these methods are unsuitable for imaging adults. Intubation methods developed for imaging anesthetized adult zebrafish (Castranova et al., 2022; Xu et al., 2015) informed the design of a modified and miniaturized imaging chamber for adult *D. cerebrum*. Given that *D. cerebrum* remains small and transparent at the adult stage, it is feasible to utilize standard confocal or multi-photon microscopy to perform whole-animal *in vivo* imaging without the need of surgical manipulation to expose regions of interest. Imaging chamber systems that facilitate mounting and positioning of adult *D. cerebrum* were designed to be used with standard upright (Fig. 1A-D) or inverted microscopes (Fig. 1E-K). These imaging chamber systems allow for *in vivo* imaging of adult *D. cerebrum* with or without anesthesia.

The imaging chamber for the inverted microscope consists of two major parts (Fig. 1B). One is the base, designed to have a footprint that will fit into any regular microscope stage insert that accepts a standard 96-well plate or a Tokai Hit STX-E series stage insert. The other is a removable concave-shaped insert with a no.1 glass coverslip adhered to the bottom with RTV silicone sealant, creating a water-tight imaging window on which the fish is mounted. This glass bottom insert fits the stage base without adhesive and ensures that any accidental over-extension of the objective does not break the glass leading to a damaged objective and water leaking onto the microscope. This base and its glass bottom insert are fitted with components to hold the inflow and outflow tubes as indicated in Fig. 1C.

Fish naturally lie on their lateral surface when excess water is removed. To mount fish in any desired orientation, wedge-shaped silicone spacers were used to position the fish prior to encasing in agarose. The silicone spacers are pieces cut from a silicone sheet. In preparation for *in vivo* time-lapse imaging, fish were completely embedded under low-melt agarose after an intubation tube was inserted into the mouth (Fig. 1C,D). By using this imaging setup, adult *D. cerebrum* in a stationary position were maintained without anesthesia for several hours, after which the fish appeared to be healthy and unaffected when they were unmounted*.* Full recovery was achieved thus far in >30 fish immobilized by using the described mounting and intubation method for at least 1 h; at least ten fish were successfully recovered after being immobilized for at least 3 h.

A similar imaging chamber design was used for the upright microscope; however, in this case the chamber only has one base piece (Fig. 1E,F). A glass slide permanently adhered in the center hole of the chamber allowed specimen illumination from below the fish and assisted in visually locating a region of interest (Fig. 1G). To immobilize fish for *in vivo* time-lapse imaging, agarose embedding similar to that described above when using an inverted microscope was used. To aid positioning the fish with its dorsal surface facing up, a mold (Fig. 1H) was designed to cast a silicone platform on which fish of different sizes can be placed. Fig. 1I shows the cast silicone platform whose finger-like projections on the right protrude to a higher level than those on the left in order to cradle and position a larger fish. An example of an intubated and mounted fish on this silicone platform is shown in Fig. 1J,K.

**Fig. 2.**
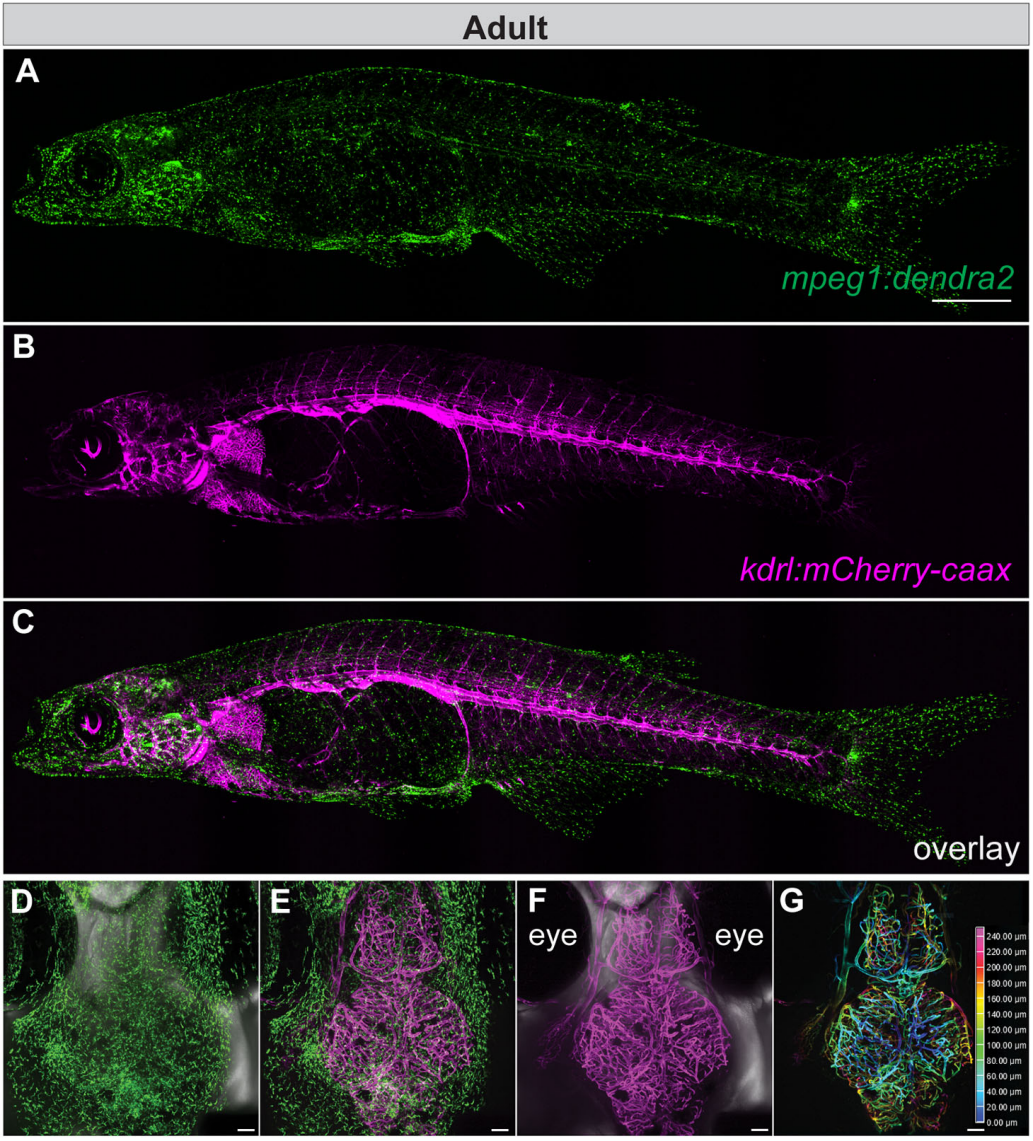
***In vivo* imaging of endothelial cells, microglia, and macrophages in transgenic adult *D. cerebrum*.** (A-C) Lateral view maximum intensity projection (MIP) of 3-month-old adult female *Tg(mpeg1:dendra2, kdrl:mCherry-caax)* fish. Anterior is to the left and dorsal is up. Images were acquired using a 4× objective NA 0.2; *z*-stack confocal images were acquired at 12.5 µm *z*-steps for a total of 825 µm. Scale bar: 1 mm. (D-G) Dorsal views of the head of a 2-month-old female *Tg(mpeg1:dendra2, kdrl:mCherry-caax)* fish. Anterior to the top. Images were acquired using a 10× Plan Apo Lambda objective NA 0.45; *z*-stack confocal images were acquired at 2.5 µm *z*-steps for a total of 250 µm. Scale bars: 100 µm. (D) Bright-field and green channel [*Tg(mpeg1:*dendra2] MIP image. (E) MIP image of merged red and green channels [*Tg(mpeg1:dendra2, kdrl:mCherry-caax)*]. (F) Bright-field and red channel [*Tg*(*kdrl:mCherry-caax)*] MIP image. (G) Color coding of the *z*-position in F and depth of focus are indicated on the scale, ranging from 0-240 µm. See Movie 2 for the corresponding 3D-rendered movie. Expression of *kdrl:mCherry-caax* is shown in magenta; expression of *Tg(mpeg1:dendra2)* is shown in green.

To test the utility and stability of the imaging system, innate immune cells, such as macrophages and microglia, were imaged as they are highly dynamic and display fine cell projections. The Tol2 transposon system (Suster et al., 2009) was used to generate a transgenic *D. cerebrum* line, in which the Dendra2 fluorescent protein is expressed under the control of the zebrafish *mpeg1* promoter*.* The zebrafish *mpeg1* promoter has previously been characterized and shown to drive transgene expression in macrophage-lineage cells (Ellett et al., 2011), including microglia (Ferrero et al., 2018), as well as in a subpopulation of B lymphocytes in adult zebrafish (Ferrero et al., 2020). In *D. cerebrum*, the zebrafish *mpeg1* promoter appeared to drive expression of the fluorescent reporter in cells with a distribution resembling that of macrophages and microglia, which were scattered throughout the entire animal (Fig. 2A and Fig. S1A) as well as in the brain (Fig. 2D and Fig. S1D) at both larval and adult stages*.* Whether *mpeg1* drives gene expression in B cells remains to be determined and is beyond the scope of this study.

To facilitate location registration at a specific region within a 3D space for longitudinal imaging, location markers were generated by fluorescently labeling tissues whose position would be relatively static in adult fish. This allowed for the same specimen to be repeatedly mounted and imaged at the exact same location during different imaging sessions. Using the zebrafish *kdrl* (*flk1*) promoter that has previously been characterized (Choi et al., 2007), a second transgenic *D. cerebrum* line, *Tg(kdrl:mCherry-caax)*, was generated. Based on the expression pattern of the transgene, the zebrafish *kdrl* promoter drives expression of *mCherry-caax* in endothelial cells in both larval (Fig. S1B,E and Movie 1) and adult *D. cerebrum* (Fig. 2B,F). A double transgenic *Tg(mpeg1:dendra2, kdrl:mCherry-caax)* line was generated by crossing *Tg(mpeg1:dendra2)* with *Tg(kdrl:mCherry-caax)* (Fig. 2C,E and Fig. S1C,F). Analysis of the *z*-plane images in Fig. 2F indicated that the brain can be imaged to a depth of at least 240 µm (Fig. 2G, Movie 2).

**Fig. 3.**
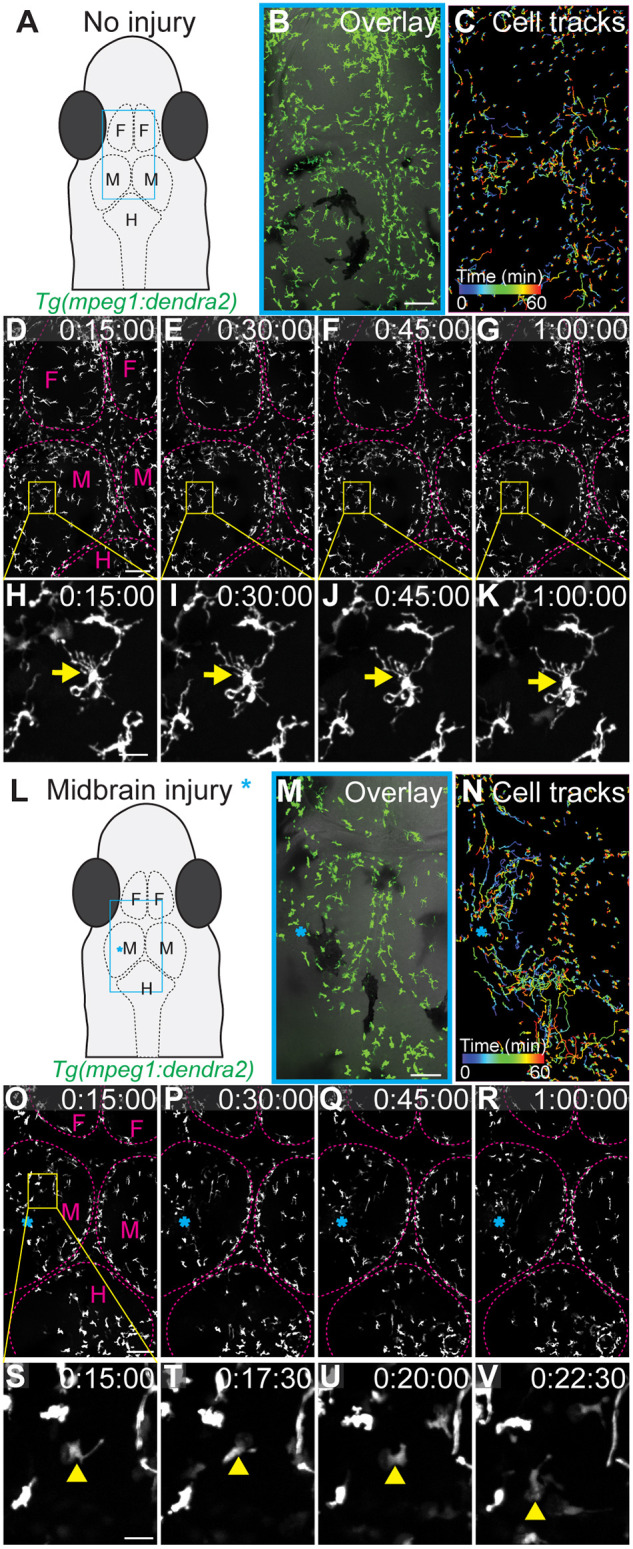
***In vivo* time-lapse imaging of microglia and/or macrophages in the brain of an immobilized and intubated adult transgenic *D. cerebrum.*** (A) Schematic representation of the head of an adult fish, showing the approximate imaging area (blue rectangle) depicted in B-G. (B) Maximum intensity projection (MIP) of the fluorescent signal in the brain of a 3-month-old female *Tg(mpeg1:dendra2)* fish superimposed with the corresponding bright-field image. The image was acquired using a long working distance 25× silicone objective NA 1.05; *z*-stack confocal images were acquired at 1 µm *z*-steps for a total of 57 µm. Two image tiles were stitched together. The same area was imaged in D-G. Scale bar: 100 µm. (C) Tracking of individual cells positive for *mpeg1:dendra2* over time. Tracks are color coded based on time. (D-G) MIP images of selected time points from a time-lapse movie. Brain regions are surrounded by dotted lines (magenta). Boxed areas are shown magnified in H-K. Scale bar: 100 µm. See Movie 3 for the full time-lapse movie. (H-K) Magnified images of the boxed areas in D-G. Arrows indicate a stationary microglia/macrophage of ramified morphology. Scale bar: 20 µm. See Movie 4 for the full time-lapse movie. (L) Schematic representation of the head of an adult fish showing the approximate imaging area (blue rectangle) depicted in M-R. The stab wound inflicted to the midbrain is indicated by a blue asterisk in L-R. (M) MIP of the brain of a 3-month-old female *Tg(mpeg1:dendra2)* fish superimposed with the corresponding bright-field image. Confocal *z*-stack images were acquired at 1 µm *z*-steps for a total of 30 µm. Three image tiles were stitched together. The same area was imaged in O-R. Scale bar: 100 µm. (N) Tracking of individual cells positive for *mpeg1:dendra2* over time. Tracks are color coded based on time. (O-R) MIP images of selected time points extracted from the time-lapse movie. Brain regions are surrounded by dotted lines (magenta). Boxed areas are shown magnified in S-V. Scale bar: 100 µm. See Movie 5 for the full time-lapse movie. (S-V) Magnified images of the boxed area in O. Selected frames from a time-lapse movie, timepoints indicated on the top of the frame. Arrowheads indicate a migrating microglia/macrophage of amoeboid morphology. Scale bar: 20 µm. See Movie 6 for the full time-lapse movie. F, forebrain; M, midbrain; H, hindbrain. Expression of *Tg(mpeg1:dendra2)* is shown in green. Time in D-K and O-V is shown in 24-h format.

In most vertebrate animal models, imaging cells within the brain is challenging since the brain is an optically less accessible tissue and often requires surgical manipulation to create an imaging window. Furthermore, microglia are highly susceptible to activation, making it imperative to image these cells without or with only little physical disturbance. In addition to being small, *D. cerebrum* lack dorsal skull bones (Schulze et al., 2018), thereby allowing direct confocal imaging of the brain by positioning the dorsal surface of the head facing the objective. To facilitate imaging of dynamic cellular processes displayed by highly motile immune cells, an inverted compound microscope with a spinning disk confocal head, combined with a Piezo-Z drive to achieve a higher *z*-stack acquisition speed was used (Lam et al., 2014). *z*-Stack spinning disk confocal time-lapse imaging of adult *Tg(mpeg1:dendra2) D. cerebrum* was performed using a 25× silicone long-working distance objective (Fig. 3). Fluorescently labeled immune cells were observed scattered in different regions of the brain under normal conditions (Fig. 3A,B). Imaris software was used to track the migration of individual cells (Fig. 3C). The distribution (Fig. 3D-G, Movie 3) and ramified morphology of these fluorescently labeled cells (Fig. 3H-K, Movie 4) was observed clearly. Since wounding studies are often used to study the behavior of innate immune cells, macrophages and/or microglia were visualized after inflicting a wound to the brain of adult *D. cerebrum*. A stab wound to the midbrain region (Fig. 3L,M) resulted in chemotactic recruitment of these fluorescently labeled immune cells to the wound site (Fig. 3N,R, Movie 5). Compared to the cells in the non-injured brain, these chemotactic cells display a less-ramified and more-amoeboid morphology (Fig. 3S-V, Movie 6).

**Fig. 4.**
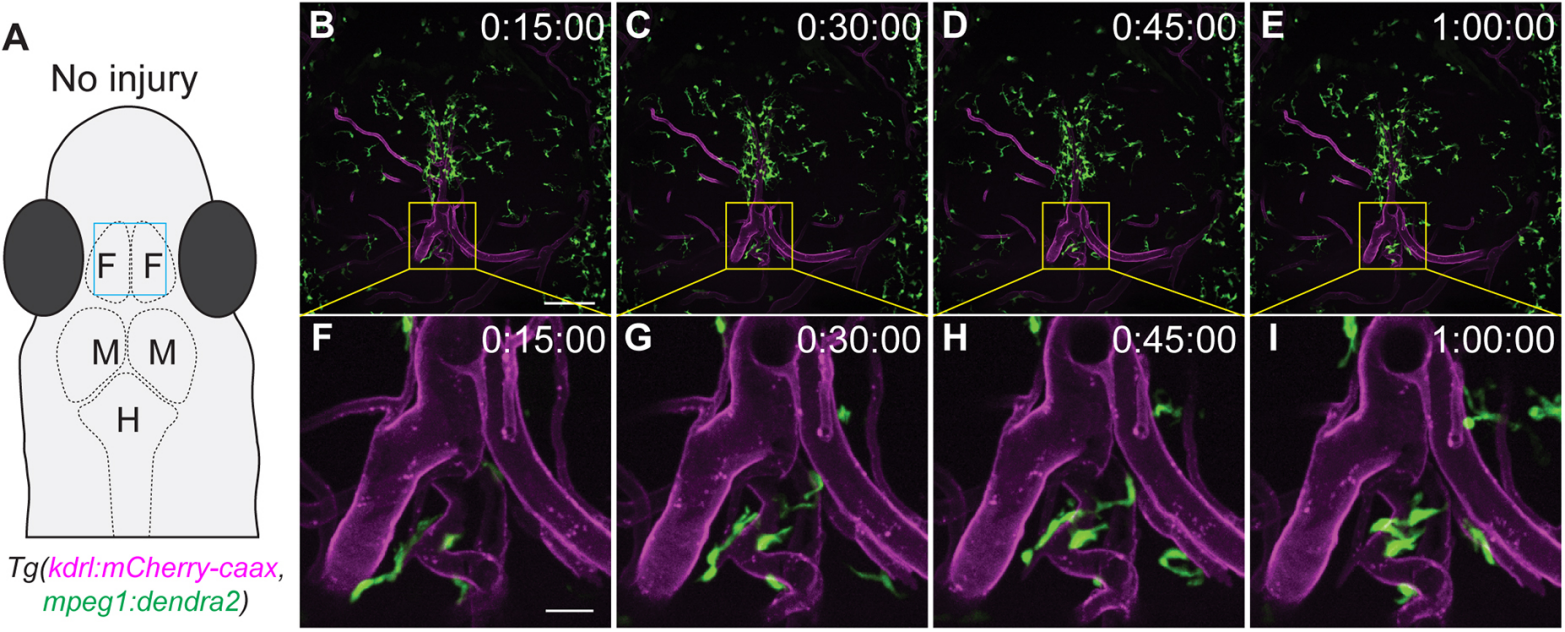
***In vivo* imaging of endothelial cells, microglia, and macrophages in adult double transgenic *Tg(mpeg1:dendra2, kdrl:mCherry-caax*) *D. cerebrum*.** (A) Schematic representation of the head of an adult fish showing the approximate imaging area (blue rectangle) depicted in B-E. F, forebrain; M, midbrain; H, hindbrain. (B-E) Maximum intensity projection of the brain of a 2-month-old female adult *Tg(mpeg1:dendra2, kdrl:mCherry-caax) D. cerebrum.* Images were acquired using a 25× silicone objective, NA 1.05. Confocal *z*-stack images were acquired at 1 µm *z*-steps for a total of 29 µm. Boxed areas are shown magnified in F-I. Scale bar: 100 µm. See Movie 7 for the full time-lapse movie. (F-I) Magnified images of the boxed area in B-E. Scale bar: 20 µm. See Movie 8 for the full time-lapse movie. Time in B-I is shown in 24-h format. Expression of *kdrl:mCherry-caax* is shown in magenta; expression of *Tg(mpeg1:dendra2)* is shown in green.

**Fig. 5.**
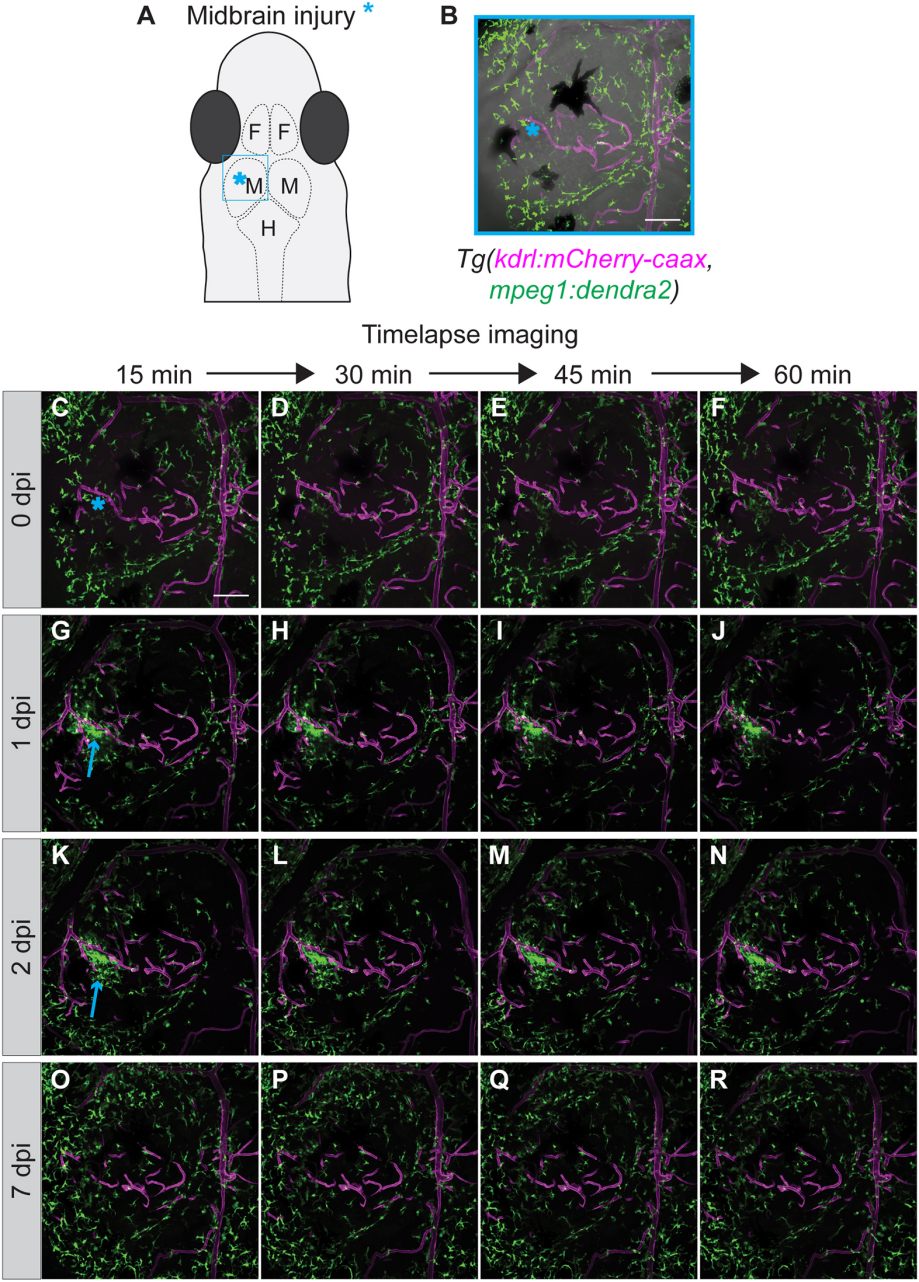
**Longitudinal time-lapse imaging of microglia and/or macrophage response to a brain stab injury in adult double transgenic *Tg(mpeg1:dendra2, kdrl:mCherry-caax) D. cerebrum.*** (A) Schematic representation of the head of an adult *D. cerebrum* showing the approximate imaging area (blue rectangle) depicted in B-R. The stab wound inflicted to the midbrain is indicated by a blue asterisk. F, forebrain; M, midbrain; H, hindbrain. (B) Maximum intensity projection (MIP) merged with a bright-field image of the brain of a 2-month-old female fish. (C-R) MIP overlay time-lapse images of the area shown in B at 0-, 1-, 2- and 7-days post injury (dpi). Imaging of the same region of interest was performed for 1 h each day at the time points indicated. Selected frames from each imaging session were extracted from the time-lapse movies. Images were acquired using a 25× silicone objective NA 1.05. Confocal *z*-stack images were acquired at 1 µm *z*-steps for a total of 60 µm. Expression of *kdrl:mCherry-caax* is shown in magenta; expression of *Tg(mpeg1:dendra2)* is shown in green. Blue asterisks indicate the stab wound inflicted to the midbrain; arrows indicate accumulation of microglia and/or macrophages at the site of injury. All scale bars: 100 µm.

Innate immune cell recruitment to a wound site is the first phase of a complex response to injury. To visualize subsequent phases, longitudinal imaging of the wound site over several days or even weeks in the same animal is beneficial. To achieve this, the *D. cerebrum* double transgenic *Tg(mpeg1:dendra2, kdrl:mCherry-caax)* adult fish was utilized. Image acquisition parameters for capturing high resolution, *in vivo* time-lapse images of the double transgenic fish were established. Sequential acquisition of Dendra2 and mCherry-caax signals was performed using a 25× silicone long-working distance objective. Interactions between immune cells and endothelial cells were observed in *z*-stack time-lapse movies of these double transgenic fish (Fig. 4A-I, Movies 7, 8). Next, the unique and identifiable patterns displayed by blood vessels from the *kdrl:mCherry-caax* fluorescent endothelial cells were utilized as registration marks for specific 3D positions. By using this registration as a guide, imaging experiments were performed on the same adult fish and at the exact same position over the course of 1 week (Fig. 5). A stab wound injury was made in the midbrain region of the fish (Fig. 5A,B) followed by *in vivo* time-lapse imaging (Fig. 5C-F). The fish was then imaged at 1 day post injury (dpi) (Fig. 5G-J), 2 dpi (Fig. 5K-N) and 7 dpi (Fig. 5O-R). After each imaging session, the fish was unmounted, allowed to briefly recover, then placed back in the fish facility between timepoints. The same position, in all three planes, could be visually confirmed based on the pattern of the surrounding endothelial cells. By using this approach, an initial recruitment of microglia and/or macrophages to the injured tissue immediately after injury was observed (Fig. 5C-F); followed by an accumulation of these immune cells in the injured tissues at 1 dpi and 2 dpi (Fig. 5G-N); and a subsequent resolution of the immune response by 7 dpi (Fig. 5O-R). The timing of the immune resolution phase was dependent on the severity of the tissue injury, as larger or deeper wounds could take upwards of 2 weeks to resolve (data not shown). This stab wound injury model and the longitudinal imaging technique demonstrate the feasibility of using adult *D. cerebrum* for wound healing and tissue-repair studies in the brain.

**Fig. 6.**
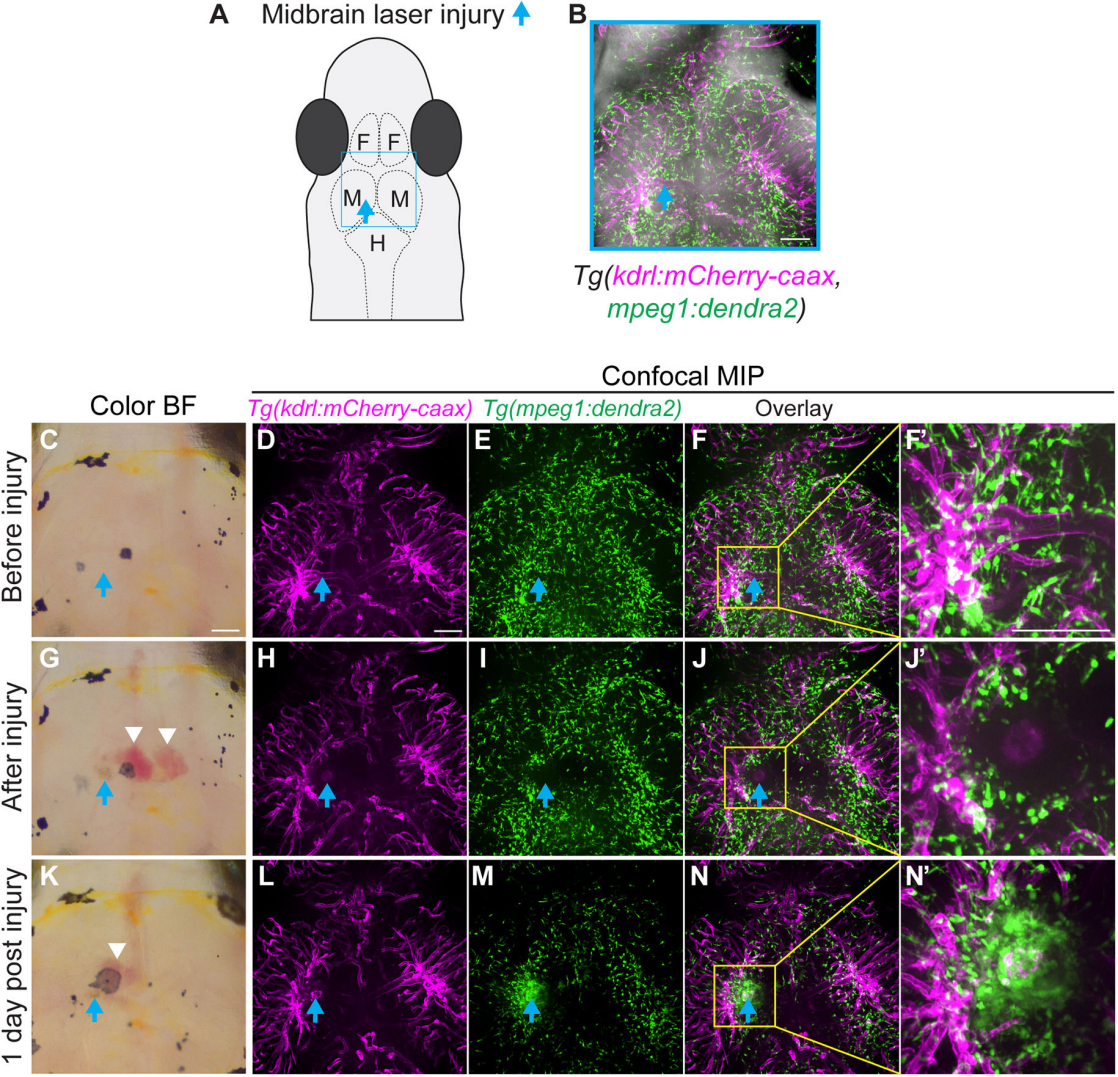
**Longitudinal imaging of the microglia and/or macrophage response to two-photon (2P) laser injury in double transgenic *Tg(mpeg1:dendra2, kdrl:mCherry-caax****)* **adult *D. cerebrum.*** (A) Schematic representation of the head of an adult *D. cerebrum* showing the approximate imaging area (blue rectangle) depicted in B-N. The location of the 2P laser wound target is indicated by the blue arrow. Laser wounding was achieved using a 2P laser (785 nm at 50% power for 5 s), targeting a 15×15 µm blood vessel region within the midbrain at a depth of ∼285 µm from the surface of the dorsal epithelial layer. F, forebrain; M, midbrain; H, hindbrain. (B) Maximum intensity projection (MIP) image merged with a bright-field image of the brain of an 8-month-old female *Tg(mpeg1:dendra2, kdrl:mCherry-caax)* fish. Approximately the same area was imaged in C-N. (C-N) Images taken before (C-F), immediately after (G-J) and 1 day (K-N) after laser wounding. Boxed areas in F, J and N are shown magnified in F’,J’ and N′, respectively. Color bright-field (BF) images are shown in C, G and K. Confocal MIP images are shown in D-F, H-J and L-N. Blue arrows indicate the location of the 2P laser wound target, white arrowheads indicate areas of hemorrhage in response to laser wounding. Images were acquired using a 20× objective NA 0.75. Confocal *z*-stack images were acquired at 1.8 µm *z*-step for a total of 149 µm. Expression of *kdrl:mCherry-caax* is shown in magenta; expression of *Tg(mpeg1:dendra2)* is shown in green. All scale bars: 100 µm.

Sterile neuroinflammation occurs in the absence of pathogens, plays a key role in the progression of neurodegenerative diseases, and is responsible for the development of secondary injury in multiple brain conditions (Banjara and Ghosh, 2017; Chen and Nunez, 2010). One of the most-severe subtypes of stroke, intracerebral hemorrhage (ICH), often leads to sterile neuroinflammation and occurs when ruptured blood vessels cause bleeding inside the brain. Neuroinflammation after hemorrhage contributes to brain damage and impacts potential repair (Taylor and Sansing, 2013; Tschoe et al., 2020). To test the feasibility of using this model to study sterile injury and brain hemorrhage, 2P laser-induced injury was used. A 2P laser allows for precise temporal and spatial control when generating tissue damage. As a proof of principle experiment, 2P laser injury on double transgenic *Tg(mpeg1:dendra2, kdrl:mCherry-caax)* adult *D. cerebrum* was performed. Using expression of *kdrl:mCherry-caax* as a guide, 2P laser injury was performed by targeting a 15×15 µm blood vessel region within the midbrain, at the depth of ∼285 µm from the surface of the dorsal epithelial layer (Fig. 6A,B). As seen in the bright-field images taken before (Fig. 6C) and after 2P-induced injury to the blood vessel (Fig. 6G,K), bleeding into the surrounding tissue was observed. Blood vessel damage was confirmed by comparing the mCherry-caax signal before (Fig. 6D,F) and immediately after 2P injury (Fig. 6H,J), as well as at 1 dpi (Fig. 6L,N). Furthermore, a robust recruitment of immune cells to the wound area was observed by comparing the Dendra2 signal after injury (Fig. 6I) and at 1 dpi (Fig. 6M). Overall, the methods for positioning and immobilization described here have proven to be highly applicable for the longitudinal imaging of live adult *D. cerebrum.* This methodology could advance the field of disease modeling by providing a non-invasive means to visualize dynamic signaling and cellular processes in live adult vertebrate animals.

## DISCUSSION

This study demonstrates the use of adult *D. cerebrum* as a longitudinal model to study tissue repair, the immune response to brain hemorrhage and sterile neuroinflammation. As wound healing is a multistage process, the ability to perform *in vivo* imaging in individual animals over time to monitor the cellular events that occur during the healing process has the potential to provide new insights. Previous studies have shown that zebrafish larvae can be used as a model to study hemorrhagic stroke (Butler et al., 2011; Crilly et al., 2018, 2019) and cerebrovascular disease (Walcott and Peterson, 2014). Data presented here show that the *D. cerebrum* model can facilitate investigating the pathological outcomes resulting from intracerebral hemorrhage and cerebrovascular disease. This includes blood–brain barrier integrity and vascular repair, endothelial cell dynamics in vascular remodeling, and the interactions between endothelial, immune and other glial cells during the maintenance of homeostasis and after injury. Unlike the zebrafish larval model, the adult *D. cerebrum* model allows for the visualization of cellular processes in a developed animal, in which different cell types have fully matured and differentiated. Furthermore, chronic and sterile low-grade inflammation (inflammaging) develops during aging and contributes to the pathogenesis of age-related diseases (Franceschi et al., 2018). An adult animal model like *D. cerebrum* can facilitate the study of tissue repair with the confounding factor of aging. The sex of *D. cerebrum* can be identified visually (Britz et al., 2021; Schulze et al., 2018), allowing for the inclusion of sex as a biological factor in any experimental study. It can be anticipated that this experimental imaging setup can be adapted to study a host of neurological disorders and diseases. For example, traumatic brain injury can be studied by adopting injury methods established in the zebrafish model such as the application of a pressure wave (Alyenbaawi et al., 2021; Babchenko et al., 2022). Similarly, infectious diseases of the central nervous system, such as bacterial meningitis (Patterson et al., 2012), can also be explored. Additionally, prolonged and longitudinal imaging of adult *D. cerebrum* will allow for a detailed study of the reciprocal interactions among the immune and nervous systems, with periphery organs and the gut microbiota (Levraud et al., 2022; Yang et al., 2020), interactions that are increasingly proving to contribute to normal development, homeostasis and disease.

To my knowledge, this is the first study to demonstrate the feasibility of longitudinal *in vivo* imaging of adult *D. cerebrum*. Using the process described here, it is possible to complete the entire mounting procedure in <10 min. An agarose-embedded fish that is subjected to continuous *z*-stack imaging can maintain health and viability for >4 h. Upon freeing the fish from restraint, they immediately appeared lively and had a high survival rate after being placed back in the main fish system. Spinning disk confocal microscopy was used in this study to acquire time-lapse images and capture the dynamic processes occurring in immune cells, which lead to changes in their morphology and chemotactic behavior. The depth of imaging achieved varied depending on the working distance of the objective used. As shown in Fig. 2G, a 250 µm depth was reached when using a 10× objective. For *in vivo* imaging in deep tissue, multi-photon imaging could be used to increase the sampling depth. For example, 2P- and three-photon (3P)-microscopy are capable of imaging to a depth of ∼600 µm and, when longer excitation wavelengths are used, to a depth of 1.6 mm (Akbari et al., 2022; Kobat et al., 2011). The fish-mounting and -positioning method described here can − depending on experimental needs – easily be adapted to any microscopy system, including multi-photon imaging, and may be used for live imaging of other similarly sized fish models, such as juvenile zebrafish and medaka.

Low-melt agarose embedding is a method commonly used in larval zebrafish imaging to allow for the positioning and stabilization of the specimen for long term *in vivo* imaging (Lam et al., 2014). By increasing the percentage of agarose and, therefore, the stiffness of the solidified agarose gel, the position of the adult *D. cerebrum* was maintained. Intubation was also added to preserve the health of the fish throughout the entire imaging session, even when prolonged. Low dose Tricaine-S was initially included in the fish water used for intubation but it was found that the agarose was sufficient for maintaining the fish in a static position during time-lapse imaging. The quality of the imaging benefited from having the fish mounted correctly, i.e. with minimal distortion of the body and the correct intubation flow rate. The mounting method described here resulted in sufficient stability that motion correction during or after acquisition was unnecessary.

These imaging chambers and mounting procedures provide a versatile way to securely position fish for live imaging. Minor modifications of some components of the chamber may be needed to customize positioning needs. These modifications could include adjusting the curve of the intubation glass capillary tube so that it fits the correct angle of the mouth of the fish. The silicone spacers may or may not be necessary, or may be cut at different angles depending on the orientation required. By using these imaging chambers, the fish could be reproducibly oriented with its dorsal or ventral surface facing up, as well as in its true lateral position. The current imaging chamber design for both inverted and upright microscope comprises only one holder for an intubation tube and, therefore, allows mounting of only one fish at a time. For higher-throughput multiplex imaging needs, modifications of the design could be made to include multiple intubation tube holders on the same device to accommodate multiple mounted fish at the same time.

In studies with rodents, repeated restraint used as a stress-inducing method has been shown to result in alterations of immune cell behavior (Kreisel et al., 2014). Restraint has been shown to cause biochemical changes in adult zebrafish brains (Dal Santo et al., 2014). It can be expected that *D. cerebrum* also experience stress during agarose immobilization. Proper controls, such as immobilization of control fish under conditions identical to those of treatment or test groups, are needed to prevent any potential variables from being introduced by the imaging procedures alone. As an alternative, recent advances have made it possible to generate high-resolution images of neuronal activity in freely swimming zebrafish larvae by using a custom-built imaging platform (Cong et al., 2017). However, substantial modifications would be needed to accommodate the larger size and faster travel speeds of adult fish, such as *D. cerebrum*.

To repeatedly image structurally dynamic processes or motile cells in a longitudinal manner over several days or even weeks and months, the registration of an accurate position within a 3D space can be achieved by fluorescently marking relatively stationary tissues in an adult animal. This study demonstrated that endothelial cells can function as a fiducial marker to identify the same region of interest because of the distinctive and stable structure of the vasculature in adult fish. It can be anticipated that other stationary structures, such as the lymphatic system (Jung et al., 2017; Okuda et al., 2012), can also be used to register a position in 3D. Generating transgenic animals in which different cell types are labeled with distinctive fluorescent proteins opens opportunities to study a variety of biological processes, such as the interaction between different cell types.

The zebrafish has been used to study the regeneration of a number of different organs and tissue types (Gemberling et al., 2013; Marques et al., 2019). It is interesting that not all model fish species possess similar competencies with respect to regeneration. For example, compared with zebrafish, another commonly used fish species, the medaka (*Oryzias latipes*), shows a very different capacity for regeneration (Chowdhury et al., 2022; Lai et al., 2017). The regenerative capacity of *D. cerebrum* is currently unknown. However, the approach using fluorescently labeled cells combined with a fluorescent reporter and assays for regeneration, such as those utilized in zebrafish (Choe et al., 2021; Gemberling et al., 2013; Moro et al., 2013), could easily allow the assessment of the regenerative capacity of different tissues or organs within the same animal over time.

With increasing popularity of using adult *D. cerebrum* as an animal model, the mounting and positioning procedure developed here for standard microscopy will provide useful and necessary resources for the *in vivo* imaging of cellular events in adult fish. Many promoter sequences originally developed for use in the zebrafish drive faithful expression in *D. cerebrum* (Schulze et al., 2018; Penalva et al., 2018). For example, this study illustrates that both the zebrafish *mpeg1* and *kdrl* promoters drive expression of fluorescent reporters in *D. cerebrum* macrophages/microglia and endothelial cells, respectively. Owing to the small size and optical transparency of adult *D. cerebrum*, optogenetic and photopharmacological tools could be applied to modulate cell activity with high temporal and spatial resolution. *D. cerebrum* could serve an important role in filling an experimental tool gap. From now on, researchers will have the ability to overcome some of the barriers that exist in other popular animal models and image *in vivo* dynamic cellular process in an adult vertebrate – without the need for complicated microscope systems or technically challenging surgical manipulation.

## MATERIALS AND METHODS

### Fish husbandry

This study was performed in accordance with the policies of the Medical College of Wisconsin's Institutional Animal Care and Use Committee (IACUC), protocol number AUA00007421. *Danionella cerebrum* stocks were originally obtained from the laboratory of Adam Douglass (University of Utah), who established a line by crossing a captive population originating from Paul Dixon and Peter Liptrot (Bolton Museum Aquarium, Bolton, UK) with wild-caught samples purchased from The Wet Spot Tropical Fish store (Portland, OR). Adult fish ≥1 month old were fed three times a day with freshly hatched *Artemia nauplii*. Between days 5 and 11 post fertilization (dpf), fish were raised in co-culture with euryhaline rotifers (*Brachonius plicatilis*). Thereafter, they were then moved into a recirculating water system, and were fed rotifers once a day and artemia three times per day until 14 dpf. From 15-30 dpf, fish were fed three times a day with artemia. All fish were maintained on a 14-hour light/10-hour dark cycle at an average temperature of 28°C.

To encourage spawning, a ∼6 cm long opaque silicone rubber tube (inner diameter 6 mm, outer diameter 8 mm; McMaster-Carr, cat. no. 5054K812) was placed on the bottom of each tank. Embryos were collected using a turkey baster style pipette and transferred to a Petri dish. Fish-tank water was then exchanged with E3 medium. Embryos were screened for the appropriate developmental stage, placed into a 28°C incubator prior to use in experiments, or grown to maintain stocks.

### Construction of imaging chambers

Two distinct imaging chambers were designed for use on either an inverted or upright microscope. Each chamber includes elements to precisely orient the specimen, and to position and secure inflow and outflow tubes to provide a constant flow of water for intubation. All components of the chamber were printed using ProJet 3500 HDMax (3D Systems, Rock Hill, SC) and VisiJet^®^ M3-X ink (3D Systems, Rock Hill, SC) 3D printers. To facilitate the use of mounting screws for rigidly fixing components in place, threads were tapped into pre-positioned holes after parts had been printed. Files used to 3D print the imaging chambers are available upon request.

### Components required for upright and inverted imaging

#### Intubation

Used were capillary clamp screws (McMaster-Carr, cat. no. 94735A706), tower set screws (McMaster-Carr, cat. no. 94613A113) and thin wall glass capillaries (3 in, OD 1.0 mm, Filament; World Precision Instruments, cat. no. TW100F-3). Glass capillary tubes were manually pulled over a Bunsen burner flame, using forceps to achieve the desired angle and diameter. The tip was then opened with a razor blade and fire-polished before use.

#### Gravity-flow system

Used were 100-ml plastic syringes with Luer Lock Connection (McMaster-Carr, cat. no. 7510A807), on/off valves with Quick-turn fitting (McMaster-Carr, cat. no. 7033T25), a lab metalware set (Fisher Scientific, cat. no. 501042880) and soft plastic tubing (McMaster-Carr, cat. nos 5186T12 and 3774N1). A gravity-flow system was used to supply a steady stream of fish-system water to the mouth and over the gills of immobilized fish at a flow rate of∼500 µl / min. Prior to use in the gravity-flow system, fish-system water was filtered using a ≤100 µm strainer to avoid contaminants that might block of the small opening of the glass capillary.

#### Outflow system

An aspiration canister (Fisher Scientific, cat. no. NC1636543) was connected to the house vacuum system. Tubing that connected the canister to a stainless-steel tube (McMaster-Carr, cat. no. 2180T14) was held in place on the imaging chamber using a nylon screw (McMaster-Carr, cat. no. 94735A719).

#### Components specific for the inverted microscope imaging chamber

Silicone sheet (McMaster-Carr, cat. no. 1460N32) to cut the wedge-shaped spacers for fish positioning. Cover glass (40×24 mm No. 1.5; Fisher Scientific, cat. no. 12-544-CP) to make the glass bottom insert. RTV silicone sealant (McMaster-Carr, Dow Corning 732) to glue the cover glass in place (allow at least 24 h for the RTV silicone sealant to cure).

#### Components specific for the upright microscope imaging chamber

Silicone (Silicones, cat. no. INC, XP-696) to cast the specimen holding insert. Glass slide (McMaster-Carr, cat. no. 1149T11) for making the clear central portion of the imaging chamber. RTV silicone sealant (McMaster-Carr, Dow Corning 732) to glue the glass slide in place (allow at least 24 h for the RTV silicone sealant to cure).

#### Expression constructs, microinjection and generation of transgenic lines

Tol2-mpeg1:dendra2 was a gift from Anna Huttenlocher (Addgene plasmid #51462; Harvie et al., 2013). Tol2-kdrl:mCherry-caax (Addgene plasmid #194287) was made by performing a Gateway LR reaction using the LR Clonase II Plus enzyme (Thermo Fisher Scientific, cat. no. 12538120), and combining p5E-kdrl(flk1) (Choi et al., 2007), pME-mCherryCAAX (Kwan et al., 2007), p3E-polyA (Kwan et al., 2007) and pDestTol2pA2 (Kwan et al., 2007). p5E-kdrl(flk1) was a gift from Jau-Nian Chen and Sarah Childs (Addgene plasmid #78687; Choi et al., 2007). Expression of the constructs was obtained by injecting 2-3 nl of a solution containing 7 ng/µl of DNA plasmid and 12.6 ng/µl *in-vitro* transcribed (Ambion) Tol2 transposase mRNA into the cytoplasm of *D. cerebrum* embryos at cell stage 1-16. Injected F0 larvae showing mosaic expression of the transgene were then grown to adulthood. The progeny of these potential founder fish displaying strong transgene expression were selected to establish the line.

#### Fish preparation, mounting, intubation and outflow setup

Fish were not fed 2 h prior to imaging. 15.3 mM Tricaine-S (Fisher Scientific, cat. no. NC0342409) stock solution at pH 7 was made as described in The Zebrafish Book (Westerfield, 1993). Prior to mounting, 4% low-melt agarose (Genesee, cat. no. 20-104) was made with E3 and equilibrated to 42°C in a digital dry bath. The imaging chamber was prepared by placing the glass bottom insert onto the base of the chamber. The glass capillary tube used for intubation was loosely placed into its holder. Initial attempts to supply fish water for intubation relied on a peristaltic pump. Although that method sufficiently maintained the health and survival of the fish during imaging, the rhythmic flow of the water through the intubation tube introduced a motion artifact. Therefore, a gravity-flow system was used to minimize any artifact generated by a mechanical pump. The gravity-fed intubation water flow was started and all tubing was checked for any trapped air bubbles. Flow rate was adjusted to ∼500 µl / min. The water flow was then stopped with a clamp. Adult fish were lightly anesthetized by placing them in fish water supplemented with ∼200 µM Tricaine-S until they started to rotate upside down. Fish were immediately transferred to the chamber and oriented as needed under a stereomicroscope with top illumination. Excess fish water was removed before a layer of molten 4% low-melt agarose (LMA) was placed on top of the body of the fish, leaving the head uncovered. The intubation tube with water flow started was then inserted into the mouth of the fish after the agarose on top of the body had solidified. The position of the intubation tube was adjusted as needed and secured in its holder by tightening the retaining screw. Water flow was then stopped and excess water removed. The head of the fish was then embedded by placing a layer of molten 4% LMA on top of the head of the fish. Before re-starting the flow of the intubation capillary water, a small opening in the agarose above the level of the gill slits was made with forceps to allow outflow of fish water. The imaging chamber with mounted and intubated fish was then transferred to the microscope stage. The outflow tubing was attached to the suction canister to remove excess water from the chamber.

#### Image acquisition

Confocal images were acquired using a Nikon Ti2 inverted microscope equipped with a Yokogawa CSU-W1 spinning disk confocal, Prime 95B Scientific CMOS camera, 488 nm or 561 nm excitation laser with the following Nikon objectives: 4× Air 0.2 N.A., 10× Air 0.45 N.A., 20× Air 0.75 N.A. and 25× Silicone 1.05 N.A.

#### Brain wounding

For stab wounding, adult fish were anesthetized by placing them in fish water supplemented with ∼200 µM Tricaine mesylate (Tricaine-S) until they started to rotate upside down. Fish were immediately transferred to a wedge-shaped holder (Fig. 1I) and put into position. A stab wound to the brain was introduced using a tungsten needle (The McCRONE Group, cat. no. 107-2) attached to a manipulator (Narishige, cat. no. M-152). The needle was inserted into the brain at a depth of approximately 1 mm, determined by using the scale on the manipulator as a guide. For 2P laser wounding, adult fish were mounted on the upright microscope imaging chamber as shown in Fig. 1J,K. A LaVision Biotec Trimscope II double-header custom multi-photon microscope was used for laser wounding of brain endothelial cells. This was achieved with a Mai Tai HP Ti:Sapphire laser at 785 nm at 50% power and a Zeiss PlanApo 2×water 1.0 N.A. objective targeting a 15 µm×15 µm region with *kdrl:mCherry-caax* expression for 5 s using a galvometric scanner with a scan speed of 200 Hz. Bright-field dorsal color images before and after laser wounding were acquired using a stereo microscope integrated with a 10 M.P. camera (Leica S9i). Fluorescent images were taken using a Yokogawa CSU-W1 spinning disk confocal as described in the Image Acquisition section.

#### Image processing

Images were acquired and processed using Nikon NIS Elements. 3D rendering and time-lapse movies were made using Nikon NIS Elements. Additional labeling was made using ImageJ (Schneider et al., 2012). Imaris ×64 version 9.8.2 was used for cell tracking.

## Supplementary Material

10.1242/dmm.049753_sup1 Supplementary information
