# Supplementary material for: Longitudinal in vivo imaging of adult Danionella cerebrum using standard confocal microscopy
Source: Dis Model Mech. 2022 Dec 22;15(12):dmm049753. doi: 10.1242/dmm.049753 (PMC9844135; doi:10.1242/dmm.049753)
Supplement: Supplementary information [file dmm-15-049753-s1.pdf]

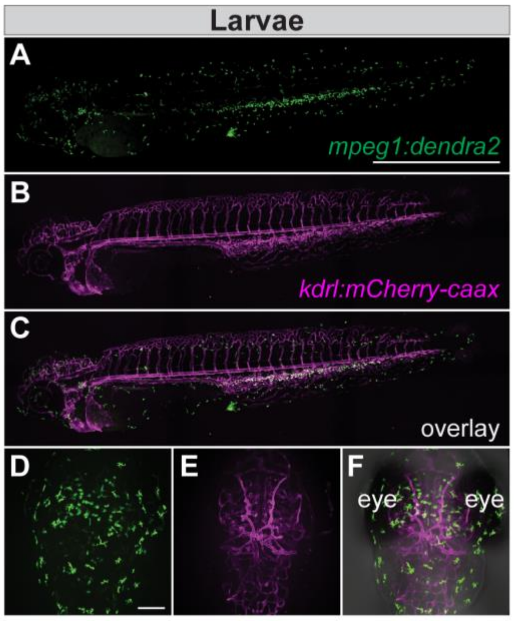

Fig. S1. *In vivo* imaging of endothelial cells, microglia, and macrophages in larval *D. cerebrum*. A maximum intensity projection of a *Tg(mpeg1:dendra2, kdrl:mCherry-caax) D. cerebrum* larvae at 3 days post fertilization (dpf). (A-C) Lateral view of a larva. Anterior is to the left and dorsal is up. Images were acquired using a 10x Plan apo lambda objective NA 0.45. *z*-stack confocal images were acquired at 2.5 µm *z*-step for a total of 357.5 µm. Six image tiles were stitched together. Scale bar = 1 mm. (D-F) Dorsal view of the larval head. Anterior to the top. Images were acquired using a 25x silicone objective NA 1.05. *z*-stack confocal images were acquired at 1 µm *z*-step for a total of 239 µm. Scale bar = 100 µm. See Supplemental Movie 1 for the corresponding 3D rendered movie.

Disease Models & Mechanisms • Supplementary information

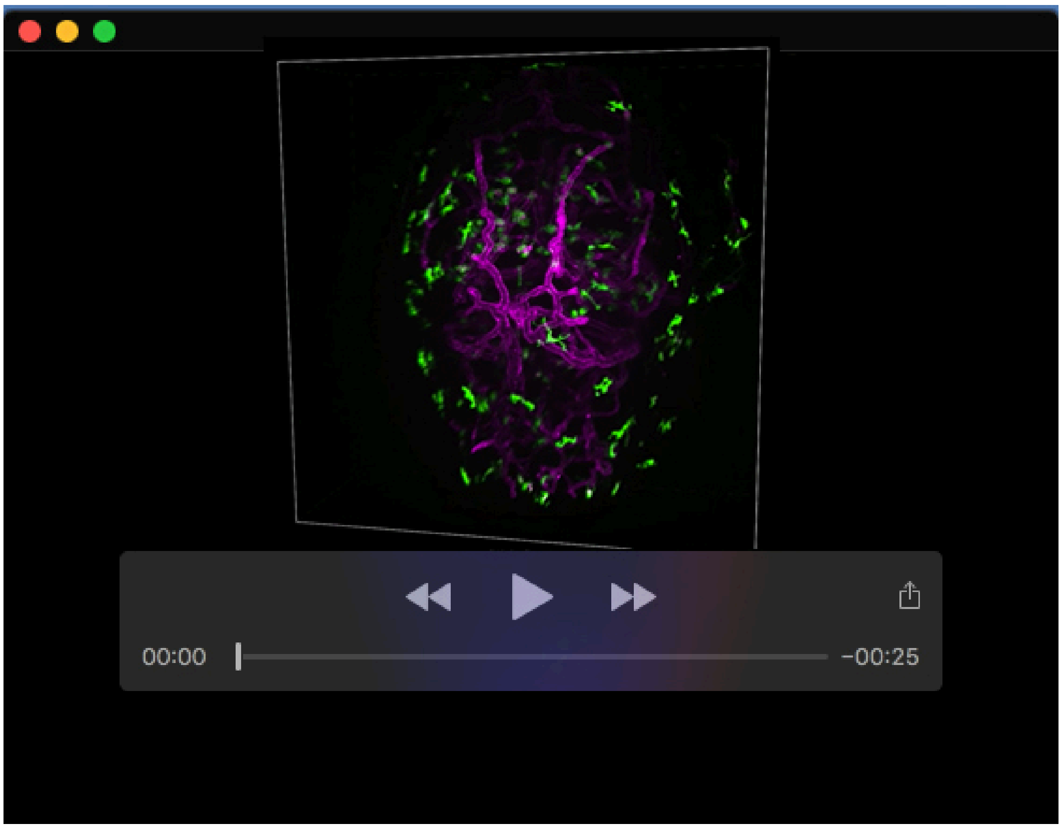

Movie 1. 3D rendering of microglia macro hages and endothelial cells in the brain of a larval *D. cerebrum*. The z-stack images of the *Tg(mpeg1:dendra2, kdrl:mCherry-caax)* double transgenic fish with fluorescent microglia macrophages (green) and endothelial cells (magenta) used to generate supplemental Figure 1F were processed into a 3D rendered movie.

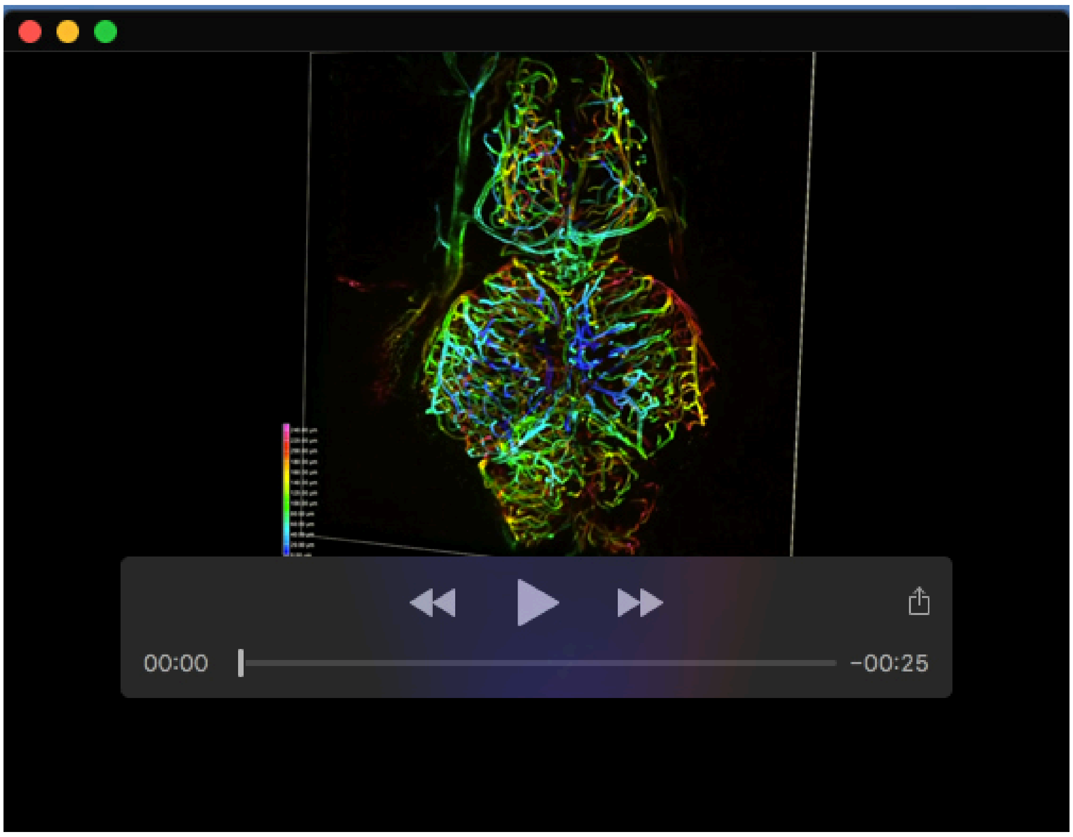

Movie 2. 3D rendering of endothelial cells in the brain of an adult *Tg(kdrl:mCherry-caax)* transgenic *D. cerebrum*. The z-stack images of the *Tg(kdrl:mCherry-caax)* transgenic fish with fluorescent endothelial cells used to generate Figure 2F were processed into a 3D rendered movie. Colors are assigned based on the depth as indicated in the scale.

Disease Models & Mechanisms • Supplementary information

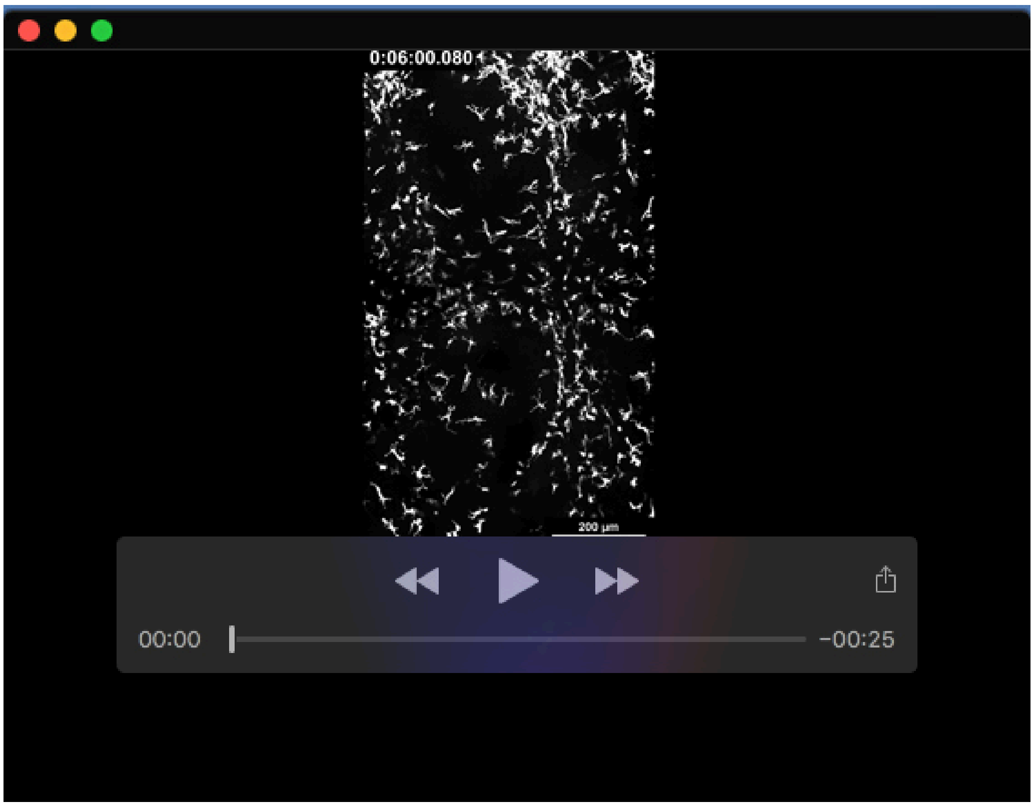

Movie 3. Time-lapse imaging of microglia/macrophages in the brain of an intubated adult *D. cerebrum*. A *Tg(mpeg1:dendra2)* transgenic fish with fluorescent microglia/ macrophages was intubated and imaged for 1 hour.

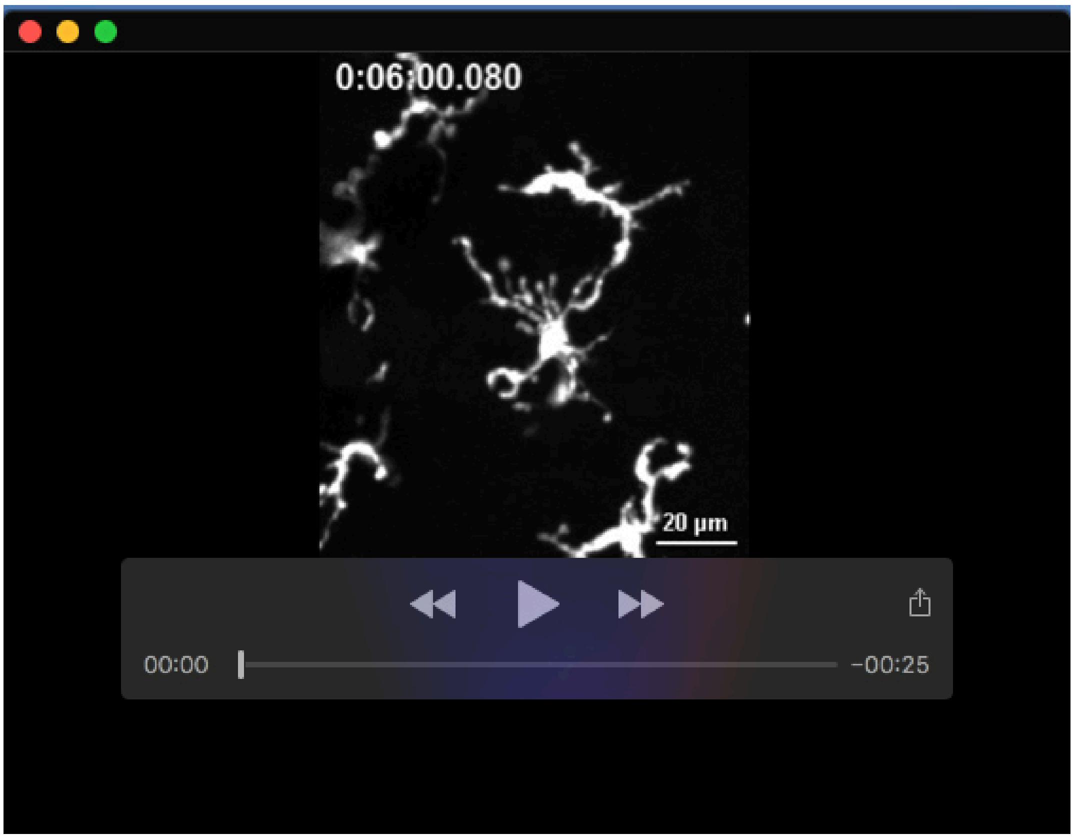

Movie 4. magnified vie of time-la se movie 3 of microglia macro hages in the brain of an intubated adult *D. cerebrum*. A *Tg(mpeg1:dendra2)* transgenic fish with fluorescent microglia macrophages was intubated and imaged for 1 hour. A portion of the field of view in time-lapse movie 2 has been magnified to highlight a subset of microglia that display a ramified morphology.

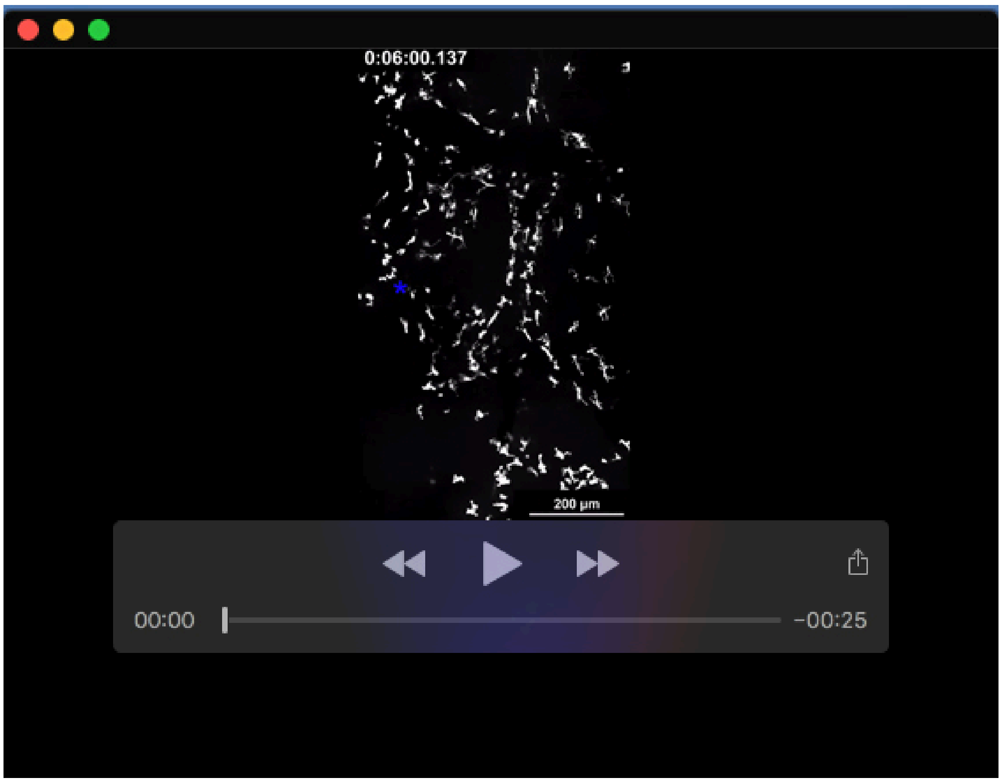

Movie 5. Time-lapse imaging of microglia/macrophage recruitment to a stab injury in the brain of an intubated adult *D. cerebrum*. A *Tg(mpeg1:dendra2)* transgenic fish with fluorescent microglia macrophages was intubated and imaged for 1 hour immediately after being subjected to a stab would injury.

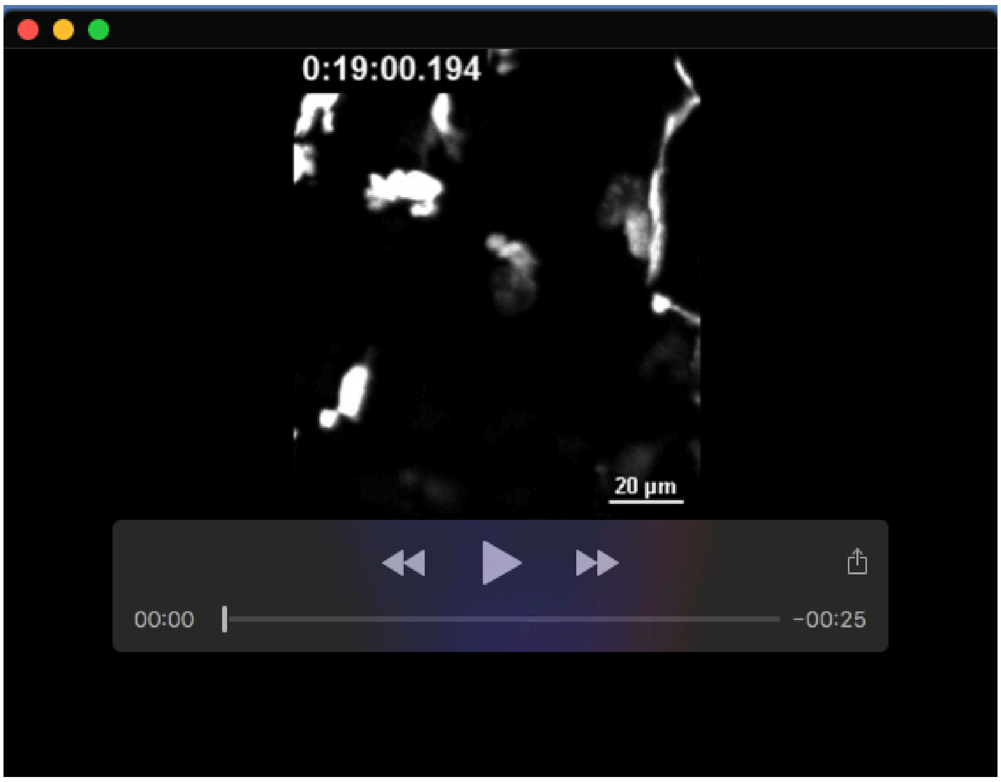

Movie 6. A magnified view of time-lapse movie 5 of microglia/macrophages of an intubated adult *D. cerebrum* that has received a stab injury to the brain. A 10 minute segment from timelapse movie 5 of a wounded *Tg(mpeg1:dendra2)* transgenic fish has been magnified to highlight a subset of microglia that display an amoeboid morphology.

Disease Models & Mechanisms • Supplementary information

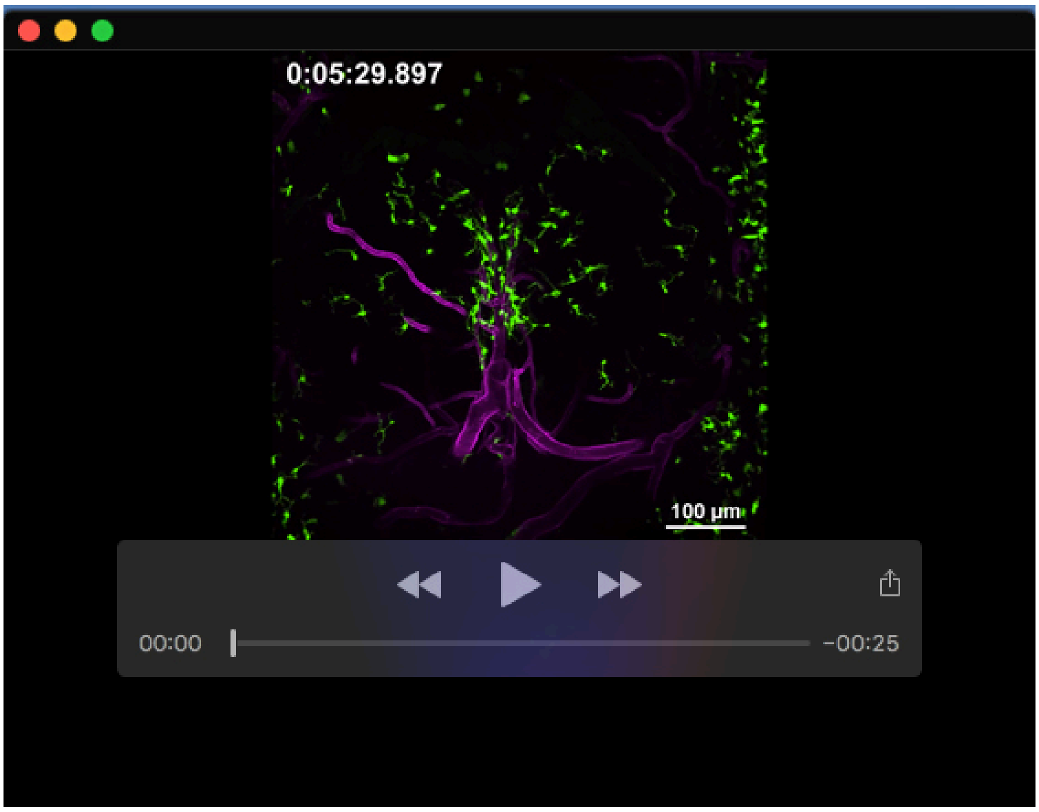

Movie 7. Time-lapse imaging of microglia/macrophages and endothelial cells in the brain of an intubated adult *D. cerebrum*. A *Tg(mpeg1:dendra2, kdrl:mCherry-caax)* double transgenic fish with fluorescent microglia macrophages (green) and endothelial cells (magenta) was intubated and imaged for 1 hour.

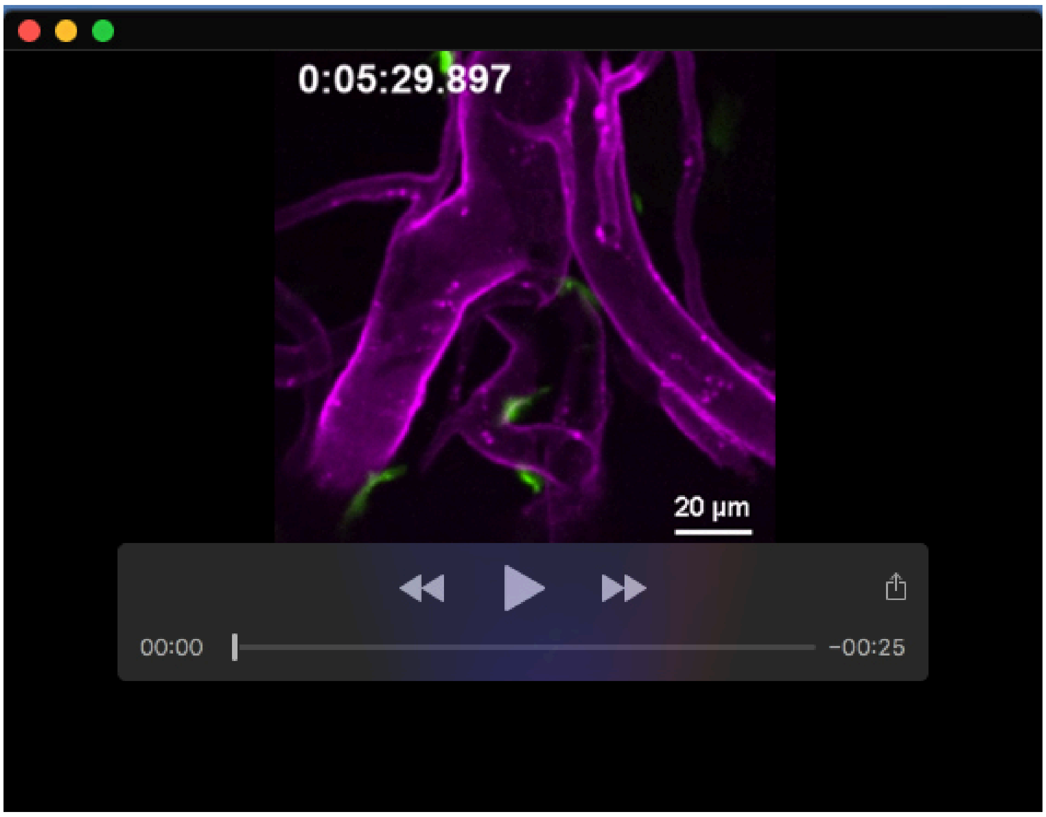

Movie 8. A magnified view of time-lapse movie 7 of microglia/macrophages and endothelial cells in the brain of an intubated adult *D. cerebrum*. A *Tg(mpeg1:dendra2, kdrl:mCherry-caax)* double transgenic fish with fluorescent microglia macrophages (green) and endothelial cells (magenta) was intubated and imaged for 1 hour.

Disease Models & Mechanisms • Supplementary information
